# Increased aqueous flare is associated with thickening of inner retinal layers in eyes with retinitis pigmentosa

**DOI:** 10.1038/srep33921

**Published:** 2016-09-22

**Authors:** Yosuke Nagasaka, Yasuki Ito, Shinji Ueno, Hiroko Terasaki

**Affiliations:** 1 Department of Ophthalmology, Nagoya University Graduate School of Medicine, 65 Tsuruma-cho, Showa-ku, Nagoya, Japan

## Abstract

Retinitis pigmentosa(RP) is a hereditary retinal disease that causes photoreceptor, outer retinal, degeneration. Although the pathogenesis is still unclear, there have been numerous reports regarding inner retinal changes in RP eyes. The aim of this study is to retrospectively evaluate the changes in the thicknesses of different retinal layers of RP eyes, and its association with aqueous flare, which is used for measuring the intensity of intraocular inflammation. A total of 125 eyes of 64 patients with RP and 13 normal eyes were studied. The thicknesses of total neural retina,nerve fiber layer(NFL),ganglion cell layer(GCL),inner plexiform layer(IPL),inner nuclear layer(INL),outer layers and foveal thickness were measured in the optical coherence tomographic images. Aqueous flare was measured with a laser flare-cell meter. The associations between those parameters, visual acuity and visual field were determined in RP eyes using multivariate analysis. The results of this study showed the significant thickening of NFL, GCL and INL, the significant thinning of outer layers and the association of them with increased aqueous flare, whereas NFL and INL thickening associated with outer retinal thinning. These results can suggest the involvement of intraocular inflammation in the pathogenesis of inner retinal thickening as a secondary change following outer retinal degeneration.

Retinitis pigmentosa (RP) is a hereditary retinal disease that causes photoreceptor degeneration. The worldwide prevalence of RP is about 1 in 4000, and the disease is a major cause of visual disturbance and blindness^1^.

The treatments, such as artificial retinas, or transplantation of photoreceptor cells or retinal pigmented epithelium (RPE), have been proposed^2^,^3^,^4^,^5^,^6^. When deciding the indication for such treatments, the status of the inner retina is important for RP patients, because it could be a main factor which determine the visual function after treatment.

There are a number of studies about the inner retinal change of RP. Using Optical coherence tomography (OCT), which can gives high quality imaging of retinal structures, recently some articles have reported that there are inner retinal changes including nerve fiber layer (NFL) thickening in eyes of RP patients^7^,^8^,^9^. However, the mechanism that causes the inner retinal change is still unclear.

On the other hand, although the pathophysiology of RP has not been established, there have been some reports about intraocular inflammation in RP eyes, which might be related to its pathophysiology^10^,^11^,^12^,^13^. Increased aqueous flare in RP eyes compared to normal eyes was reported two decades ago^13^, and recently chronic inflammation in RP eyes, such as cells in the anterior vitreous cavity and the increased level of proinflammatory cytokines and chemokines in the aqueous humor and vitreous, was also reported^12^.

We hypothesized the association between the inner retinal change and the intraocular inflammation. In order to investigate this relationship, we measured the aqueous flare value, as an intraocular inflammatory parameter, in RP eyes using laser flare-cell meter. We also determined its association with the thicknesses of different retinal layers measured using OCT, best-corrected visual acuity (BCVA) and visual field in this study.

## Results

The aqueous flare value was 7.9 ± 3.5 pc/ms in RP eyes without cystoid macular edema (CME), 12.0 ± 7.8 pc/ms in RP eyes with CME, and 3.5 ± 1.0 pc/ms in eyes of controls. The differences between any pair of the three groups were significant (ANOVA: *P* < 0.001, RP eyes with CME vs RP eyes without CME: *P* < 0.006, RP eyes without CME vs eyes of controls: *P* < 0.001).

The BCVA in controls was significantly better than that in RP eyes with and without CME (*P* = 0.031 and *P* = 0.01, respectively). The BCVA in RP eyes with and without CME were not significantly different (*P* = 0.99). The differences in age, IOP, and refractive error between RP eyes without CME, RP eyes with CME, and controls were not significant (all *P* > 0.05; Table 1).

**Figure 1:**
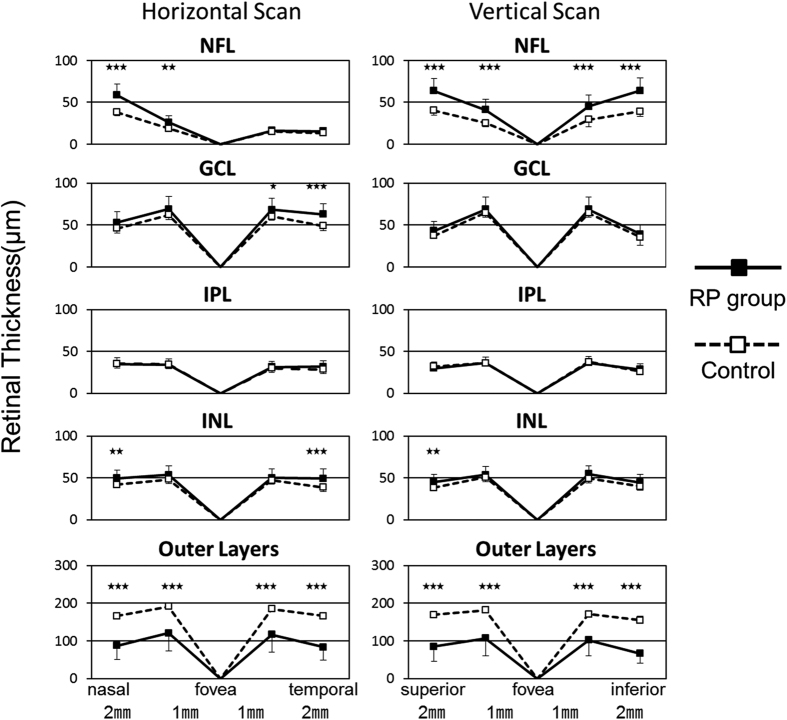
Thickness of different retinal layers at 8 points.

The thicknesses of each retinal layer in RP eyes at different measured points are shown in Fig. 1. The mean thicknesses of each retinal layer in RP eyes and controls are shown in Supplementary Table S1. The thickness of total neural retina of RP eyes was 279.9 ± 41.9 μm which was significantly thinner than that of controls of 330.2 ± 11.8 μm (*P* < 0.001). The NFL thickness of RP eyes and controls were 41.3 ± 7.1 μm and 27.6 ± 2.1 μm respectively. The GCL thickness of RP eyes and controls were 59.3 ± 9.7 μm and 52.5 ± 2.5 μm respectively. NFL and GCL were significantly thicker in RP eyes than controls (*P* < 0.001 and *P* = 0.01 respectively). The IPL thickness of RP eyes and controls were 33.3 ± 4.9 μm and 33.0 ± 2.2 μm respectively, and these were not significantly different (*P* = 0.84). The INL thickness of RP eyes and controls were 50.3 ± 7.2 μm and 44.4 ± 2.3 μm respectively. INL was significantly thicker in RP eyes than controls (*P* = 0.004). The thickness of outer layers of RP eyes was 95.9 ± 36.5 μm which was significantly thinner than controls of 172.8 ± 7.9 μm (*P* < 0.001). The visual field scores of RP eyes without CME were 3.6 ± 3.2.

**Figure 2:**
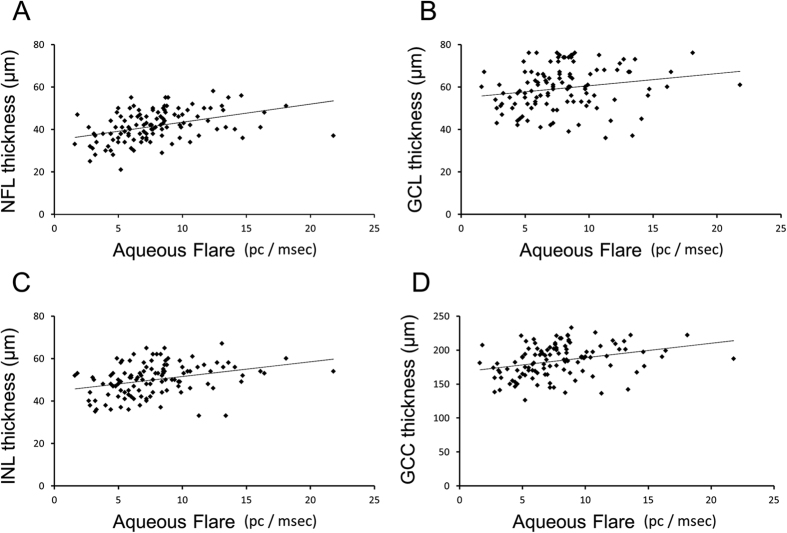
Scatter plots of the thickness of the different inner retinal layers as a function of aqueous flare. NFL: nerve fiber layer, GCL: ganglion cell layer, INL: inner nuclear layer, GCC: ganglion cell complex (GCC = NFL + GCL + IPL).

Generalized estimating equation (GEE) showed that the NFL thickness was significantly and positively associated with aqueous flare (*P* = 0.006) and negatively with the thickness of outer layers (*P* = 0.049; Fig. 2, Supplementary Table S2). The GCL thickness was significantly and positively associated with aqueous flare (*P* = 0.017; Fig. 2, Supplementary Table S2). The INL thickness was significantly associated with aqueous flare positively (*P* = 0.005; Fig. 2, Supplementary Table S2), and the thickness of outer layers negatively (*P* = 0.026). GEE showed that the thickness of ganglion cell complex (GCC = NFL + GCL + IPL) was significantly associated with aqueous flare positively (*P* < 0.001; Fig. 2, Supplementary Table S2). GEE showed that BCVA was significantly associated with the foveal thickness (*P* < 0.001; Supplementary Table S3). The factor most highly associated with the visual field score positively was the thickness of outer layers (*P* < 0.001) and negatively was aqueous flare (*P* = 0.040; Supplementary Table S4).

The intra-grader ICCs for NFL, GCL, IPL, INL and OL thickness were 0.98, 0.83, 0.85, 0.88, and 0.99 respectively. The inter-grader ICCs for the same layer thickness were 0.97, 0.82, 0.82, 0.88, and 0.99 respectively. Thus, the reproducibility of the measurements of the thicknesses of each retinal layer by the manual method was confirmed.

## Discussion

There have been a number of studies that reports the increase of aqueous flare in various retinal diseases including diabetic retinopathy, retinal vein occlusion, age-related macular degeneration^14^,^15^,^16^. In their studies, aqueous flare measured with laser flare-cell meter was reported to relate to the disease activity, and often it was described due to the inflammation. Recently, it was also reported the close relationship between intraocular inflammation and central visual function in the eyes with retinitis pigmentosa, with increased aqueous flare measured using laser flare-cell meter^17^. Thus, we thought that this non-invasive, quantitative evaluation of intraocular inflammation determined by aqueous flare value was also possible for the analysis, to investigate the relationship between the change of retinal thickness and intraocular inflammation in this study.

Our OCT results showed that NFL, GCL and INL were thicker in RP eyes than those of control eyes, whereas outer layers of RP eyes were thinner than that of control eyes. Our data also demonstrated that the aqueous flare value of RP eyes was higher than that of control eyes. These results are mostly consistent with previous studies^7^,^13^, but our study first determined significant associations between the thickening of inner retina, increased aqueous flare, and the thinning of outer retina. Based on these results, it could be considered as follows. The association in the thickening of NFL, GCL and INL with increased aqueous flare suggests that the inner retinal thickening has relationship with intraocular inflammation. The association in the thinning of outer retina and visual field loss with increased aqueous flare suggests that the intraocular inflammation occurs with the loss of photoreceptor, so that suggests the disease progression. The association in the thickening of NFL and INL with the thinning of outer layers suggests that the inner retinal thickening is a secondary change following the outer retinal degeneration.

Other OCT studies have reported changes of the inner retinal thickness in eyes of RP^7^,^8^,^9^. They mentioned mostly NFL thickening in macula or peripapillary area, however, they also reported a part of patients with the NFL thinning. The rate is different among their results and the reason for this difference is still in discussion. Because the genetic background of the patients and severity of the visual disturbance of all eyes including ours are different, the differences may be due to this.

Our results showed the significant GCL and INL thickening in RP eyes. It was reported in the previous studies that the ganglion cell counts and cell nuclei counts in INL were lower in postmortem eyes of RP patient than those of normal controls, with no differences when comparing between different hereditary types^18^,^19^. Taken together, it may be possible to guess that the cells in GCL and INL are decreased, whereas these layers are thickened in RP eyes.

Our results suggest that the inflammatory reaction is associated with the inner retinal thickening. Although the reason has been unclear, it has been proposed that axonal swelling, retinal remodeling involving neuronal migration and glial hypertrophy, or a mechanical factor as possible causes of the NFL thickening^7^. Our results can provide the connection between those inner retinal change and intraocular inflammation.

Recently, Yoshida *et al*. reported increased expression of proinflammatory cytokines and chemokines, activation of microglia in outer nuclear layer and photoreceptor apoptosis during retinal degeneration of rd10 mice^11^. They also reported suppression of these factor induced by the material which suppresses microglia^11^. This study suggests that microglia is associated with the inflammatory reaction and photoreceptor cell death. Based on this report, the associations in the thinning of the outer retina, visual field loss and increased aqueous flare could suggests that these inflammatory reactions are related to the photoreceptor cell death in eyes of RP patients. Thus, if the inflammation causes the inner retinal change in RP eyes, a treatment to suppress the inflammation could be one of the possibilities to protect the inner retina from unexpected changes. Microglia–inhibiting factors which are being explored in the treatment of various degenerative diseases of the central nervous system including multiple sclerosis, Alzheimer’s disease, and Parkinson’s disease^20^,^21^,^22^,^23^, might be helpful for the suppression of intraocular inflammation.

The degree of aqueous flare is significantly higher in patients with CME than without CME in our study. This result is the same as previous study. Recently, some studies have reported that some anti-inflammatory treatment such as dexamethasone intraocular implant treatment is effective for CME^24^,^25^. These findings can suggest that the pathogenesis of CME is also associated to the intraocular inflammation.

There have been numerous studies about the treatments that compensate for the loss of photoreceptors such as photoreceptor transplants and artificial retinas^2^,^5^. Argus II, a new type of epiretinal prosthesis, was approved by Food and Drug Administration in United States in 2013^26^. Whereas the treatment that rescues photoreceptors has been developed, inner retinal status is important for such treatments, because it depends on the presence of healthy inner retinal condition. The imaging method such as SD-OCT is a strong tool which can gives detail morphometric analysis in RP eyes to evaluate the changes of the retinal layers. Although our study provides an additional knowledge to previous investigations of inner retinal analysis of RP eyes, further study is still needed to evaluate the inner retinal status for adequate treatment.

The visual functions of the patients in our study might be relatively preserved compared with all RP patients, because they who are blind do not visit outpatient clinics periodically. This is one limitation of our study. Additionally, we analyzed small sample size, especially in normal control group. Bigger sample size should be analyzed in future investigation. The manual measurement of retinal thickness is also a limitation. However, the ICCs of each retinal layer thickness were substantial to nearly perfect (0.80–0.91) in our study. The strength of this study is that the findings of this study which has pathophysiological implication, is totally based on non-invasively evaluated clinical data.

In conclusion, our study first showed the thickening of GCL and INL layer in RP eyes in addition to NFL change previously described. Our study also showed the thinning of outer layers and increased aqueous flare, consistent with previous studies. These results are similar to other studies. However, our study first demonstrated that increased aqueous flare associates with inner retinal thickening, outer retinal thinning and visual field loss in RP eyes, and that their inner retinal thickening associates with outer retinal thinning. These results can suggest the involvement of intraocular inflammation in the pathogenesis of inner retinal thickening as a secondary change following outer retinal degeneration. These findings can provide pathophysiological implication of inner retinal change of RP eyes to this field.

## Methods

### Subjects

The medical records of 64 patients with RP who had been examined at Nagoya University Hospital from April 2012 to June 2014 were studied. There were 26 men and 38 women whose mean ± standard deviation (SD) age was 47.5 ± 15.2 years with a range of 15 to 74 years. Both eyes of one patient with Usher syndrome were also included.

Eyes with the vitreomacular traction syndrome (VMTS), macular hole, epiretinal membrane (ERM) which affect the retinal thickness, intraocular lens implanted, glaucoma, intraocular pressure higher than 21 mmHg, diabetic retinopathy, and cataracts that affected the visual acuity were excluded. Eyes after cataract surgery were removed because cataract surgery was reported to cause inflammation and affect the long-term postoperative flare value^27^. In the end, 3 of the 128 eyes of 64 patients were excluded; one eye each with a macular hole, VMTS, and cataract.

The RP eyes were divided into eyes with and without cystoid macular edema (CME) in the spectral domain (SD) OCT images. There were 104 RP eyes of 53 patients (20 men and 33 women, average age: 46.9 ± 14.4 years) without CME and 21 RP eyes of 11 patients (6 men and 5 women, average age: 50.3 ± 18.9 years) with CME (Table 1).

As controls, 13 normal fellow eyes of patients with unilateral idiopathic macular holes or unilateral retinal detachments, and 1 eye of a normal healthy subject were studied. Eyes of controls were age- and refractive error- matched to RP eyes without CME. In controls, there were 6 men and 7 women whose mean ± SD age was 50.4 ± 13.6 years with a range of 27 to 66 years (Table 1).

The protocol for this retrospective study was approved by the Institutional Review board of Nagoya University School of Medicine, and the procedures used conformed to the tenets of the Declaration of Helsinki. Informed consent was not required for this retrospective study.

### Examinations

The examinations included SD-OCT (Spectralis, Heidelberg Engineering, Heidelberg, Germany), and measurements of BCVA, intraocular pressure (IOP) with a non-contact tonometer (NT-530P; Nidek, Gamagori, Japan), aqueous flare with a laser flare-cell meter (FM-600 KOWA Company, Tokyo, Japan), and refractive error (spherical equivalent) with the KR-8900 auto Kerato-Refractometer (Topcon, Tokyo, Japan). Visual fields were determined with the Goldmann perimeter. For the Spectralis OCT images, image quality less than 15 dB was excluded. Decimal visual acuity was converted to logarithm of the minimal angle of resolution (logMAR) units for statistical analyses.

### Measurements of retinal layer thicknesses

**Figure 3:**
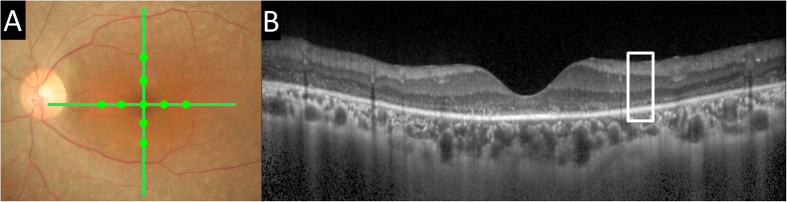
Fundus photograph and optical coherence tomographic (OCT) images of a patient with retinitis pigmentosa (RP). (**A**) Representative fundus photograph showing the locations of the 9 mm OCT scans (green lines). Green filled circle indicates the location of the measured points. (**B**) Representative OCT image. Note that the photoreceptor layer is lost.

The Spectralis OCT was used to record the cross sectional scans across the retina centered on the fovea (9 mm vertical and horizontal; Fig. 3). One hundred images were averaged with the eye tracking feature to reduce noise and to obtain clearer views of the different retinal layers.

**Figure 4:**
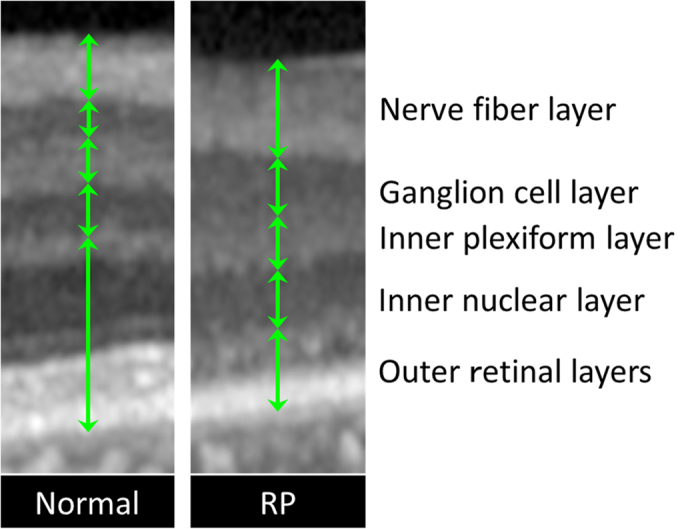
Comparison of each retinal layer thickness between eyes with retinitis pigmentosa and controls. Magnified image of the white square in Fig. 3 and corresponding area of normal control. The thickness of each layer was measured and compared.

In OCT images, the thicknesses of total neural retina, nerve fiver layer (NFL), ganglion cell layer (GCL), inner plexiform layer (IPL), inner nuclear layer (INL), and outer layers were manually measured. The measurements were made at 1 and 2 mm superior, inferior, nasal, and temporal to the fovea in the vertical and horizontal images using the built-in caliper function in the Spectralis OCT (version 5.3; Heidelberg Engineering, Heidelberg, Germany; Fig. 4). The mean thicknesses of each layer at these 8 points were used for the statistical analyses. Foveal thickness was also measured in both the horizontal and vertical images, and the mean thickness was calculated. The thicknesses of each layer were not measured in RP eyes with CME because it was difficult to make accurate measurements due to the cysts and deformation of the layers. The measurements were performed blind to the clinical information including age, sex or aqueous flare value.

### Measurements of Aqueous Flare

Aqueous flare values were measured with the FC-500 laser flare-cell meter (Kowa Company, Ltd, Tokyo, Japan). The laser flare-cell meter emits a laser beam (635 nm) into the anterior chamber and measures the backscattered light. The intensity of backscattered light is proportional to the concentration and size of proteins in the anterior chamber^28^,^29^,^30^,^31^. The accuracy and reproducibility of the flare values have been reported in several studies by different groups^28^,^29^,^30^,^31^. The coefficient of variation was less than 10%, and the measurements were independent of the examiner making measurements. In our study, more than seven measurements were obtained and averaged for each eye. The results were expressed as photon counts per millisecond (pc/ms).

### Visual field scores

We evaluated the visual field by Goldmann kinetic perimetry using the previously proposed scoring system^32^. In brief, the visual field was divided into 12 sectors by horizontal and vertical lines through the center and concentric circles of 10 and 30 degrees. In each sector, a score of 1 was assigned if the visual field of V/4e isopter was totally preserved. A score of 0.5 was assigned if the visual field was preserved in more than one-half but not the whole sector. A score of 0 was assigned if the visual field was less than one-half in each sector. In this visual field scoring system, the visual fields were graded into 24 steps from 0 to 12 in 0.5 steps.

### Reproducibility

The reproducibility of the thickness measurements was determined by intra- and inter-grader interclass correlation coefficients (ICCs). The thickness of each layer was measured in 15 randomly selected OCT images by a single grader twice for the intra-grader ICCs. For the inter-grader ICCs, each layer was measured by 2 graders independently. For both measurements, the graders were masked to the clinical characteristics of the subjects, e.g., the age, sex, normal or with retinitis pigmentosa.

### Data Analyses

The thicknesses of each retinal layer in the OCT images of RP eyes without CME were compared to those of the corresponding layers in controls using unpaired *t* tests. Age, IOP, refractive error, BCVA, and aqueous flare values in the RP eyes without CME and with CME were compared to those of controls using one-way analysis of variance (ANOVA) with Tukey’s post-hoc test.

In RP eyes without CME, GEE^33^,^34^ was performed to find the parameters that were significantly associated with the thicknesses of different retinal layers. In addition, GEE was used to find the parameters that were significantly associated with BCVA and visual field scores. The results were expressed as mean ± SD. A *P* value < 0.05 was considered statistically significant. All statistical analyses were performed using IBM SPSS 22.0 software (SPSS, Chicago, IL).

## Additional Information

**How to cite this article**: Nagasaka, Y. *et al*. Increased aqueous flare is associated with thickening of inner retinal layers in eyes with retinitis pigmentosa. *Sci. Rep.* **6**, 33921; doi: 10.1038/srep33921 (2016).

## Supplementary Material

Supplementary Information

## Figures and Tables

**Table 1:** Characteristics of controls, and RP eyes with and without CME.

	Controls	RP eyes without CME	RP eyes with CME	*P*
Number of Eyes	13	104	21	
Male/Female	6/7	20/33	6/5	^†^0.24
Age (yrs)	50.4 ± 13.6	46.9 ± 14.4	50.3 ± 18.9	^‡^0.41
IOP (mmHg)	13.6 ± 3.0	13.1 ± 2.4	13.2 ± 2.5	^‡^0.87
SERE (diopters)	−1.6 ± 2.1	−1.4 ± 3.1	−2.9 ± 3.1	^‡^0.17
BCVA (logMAR)	0.0 ± 0.0	0.39 ± 0.48	0.40 ± 0.45	^‡^0.13

RP = retinitis pigmentosa; CME = cystoid macular edema; IOP = intraocular pressure; SERE = spherical equivalent refractive error; BCVA = best-corrected visual acuity; ^†^chi-square test; ^‡^analysis of variance.
