# Supplementary material for: Increased aqueous flare is associated with thickening of inner retinal layers in eyes with retinitis pigmentosa
Source: Sci Rep. 2016 Sep 22;6:33921. doi: 10.1038/srep33921 (PMC5031997; doi:10.1038/srep33921)
Supplement: Supplementary Information [file srep33921-s1.pdf]

Supplementary information

Increased aqueous flare is associated with thickening of inner retinal layers in eyes with retinitis pigmentosa

Yosuke Nagasaka[1], Yasuki Ito[1]*, Shinji Ueno[1], Hiroko Terasaki[1],

[1]Department of Ophthalmology, Nagoya University Graduate School of Medicine, Nagoya, Japan

Supplementary Table S1: Thickness of each retinal layer of RP eyes without CME group and control group (μm)

	NFL	GCL	IPL	INL	Outer layers	Total neural retina	Foveal Thickness
**RP eyes without CME**	41.3±7.1	59.3±9.7	33.3±4.9	50.3±7.2	95.9±36.5	279.9±41.9	200.6±62.9
**Controls**	27.6±2.1	52.5±2.5	33.0±2.2	44.4±2.3	172.9±7.9	330.2±11.8	227.6±18.3
P	<0.001	0.01	0.84	0.004	<0.001	<0.001	0.12

NFL= nerve fiber layer; GCL=ganglion cell layer; IPL= inner plexiform layer; INL=inner nuclear layer; RP=retinitis pigmentosa; CME=cystoid macular edema; Total neural retina= NFL+GCL+IPL+INL+Outer layers

Supplementary Table S2: Association of covariables with the thicker retinal layers in RP eyes

Dependent Variable	Covariables	β	Standard Error	95% Wald Confidence Interval	*P*
Nerve Fiber Layer	Aqueous flare (pc/ms)	0.75	0.27	0.22 to 1.27	0.006
	Outer layers (μm)	-0.042	0.022	-0.085 to -0.000	0.049
	SERE (diopters)	0.17	0.16	-0.15 to 0.49	0.29
	Age (yrs)	-0.039	0.061	-0.16 to 0.080	0.52
Ganglion Cell Layer	Aqueous flare (pc/ms)	0.76	0.32	0.14 to 1.37	0.017
	Outer layers (μm)	0.033	0.29	-0.023 to 0.089	0.25
	SERE (diopters)	0.23	0.39	-0.53 to 0.99	0.55
	Age (yrs)	-0.085	0.075	-0.23 to 0.063	0.26
Inner Nuclear Layer	Aqueous flare (pc/ms)	0.46	0.16	0.14 to 0.78	0.005
	Outer layers (μm)	-0.043	0.019	-0.080 to -0.005	0.026
	SERE (diopters)	-0.40	0.21	-0.82 to 0.014	0.058
	Age (yrs)	0.00	0.058	-0.12 to 0.11	0.99
Ganglion Cell Complex	Aqueous flare (pc/ms)	0.81	0.23	0.37 to 1.26	<0.001
	Outer layers (μm)	0.053	0.057	-0.058 to 0.16	0.35
	SERE (diopters)	1.23	0.69	-0.13 to 2.59	0.076
	Age (yrs)	-0.21	0.15	-0.51 to 0.094	0.18

SERE= spherical equivalent refractive error

Supplementary Table S3: Association of covariables with the visual acuity in RP eyes

Dependent Variable	Covariables	β	Standard Error	95% Wald Confidence Interval	*P*
BCVA (logMAR)	Foveal thickness (µm)	-0.0050	0.00060	-0.0060 to 0.0035	<0.001
	Age (yrs)	0.0030	0.0026	-0.0020 to 0.008	0.18
	Aqueous flare (pc/ms)	0.0090	0.011	-0.012 to 0.030	0.38
	SERE (diopters)	0.0080	0.011	-0.013 to 0.030	0.46

BCVA= best-corrected visual acuity; SERE= spherical equivalent refractive error

Supplementary Table S4: Association of covariables with the visual field in RP eyes

Dependent Variable	Covariables	β	Standard Error	95% Wald Confidence Interval	*P*
Visual field score	Outer layers (µm)	0.064	0.0075	0.049 to 0.078	<0.001
	Aqueous flare (pc/ms)	-0.14	0.070	-0.28 to -0.007	0.040
	Age (yrs)	-0.024	0.022	-0.067 to 0.019	0.28
	SERE (diopters)	0.070	0.10	-0.13 to 0.27	0.49

SERE= spherical equivalent refractive error
